# Pharmacokinetics and Pharmacodynamics of a Novel Virulent Klebsiella Phage Kp_Pokalde_002 in a Mouse Model

**DOI:** 10.3389/fcimb.2021.684704

**Published:** 2021-08-16

**Authors:** Gunaraj Dhungana, Roshan Nepal, Madhav Regmi, Rajani Malla

**Affiliations:** ^1^Central Department of Biotechnology, Tribhuvan University, Kirtipur, Nepal; ^2^Adelaide Medical School, Faculty of Health and Medical Sciences, The University of Adelaide, Adelaide, SA, Australia

**Keywords:** bacteriophage, PK/PD, carbapenem-resistant infections, *Klebsiella pneumoniae*, phage therapy

## Abstract

Phage therapy is one of the most promising alternatives to antibiotics as we face global antibiotic resistance crisis. However, the pharmacokinetics (PK) and pharmacodynamics (PD) of phage therapy are largely unknown. In the present study, we aimed to evaluate the PK/PD of a locally isolated virulent novel øKp_Pokalde_002 (*Podoviridae*, C1 morphotype) that infects carbapenem-resistant *Klebsiella pneumoniae* (Kp56) using oral and intraperitoneal (IP) route in a mouse model. The result showed that the øKp_Pokalde_002 rapidly distributed into the systemic circulation within an hour *via* both oral and IP routes. A higher concentration of phage in plasma was found after 4 h (2.3 x 10^5^ PFU/ml) and 8 h (7.3 x 10^4^ PFU/ml) of administration through IP and oral route, respectively. The phage titer significantly decreased in the blood and other tissues, liver, kidneys, and spleen after 24 h and completely cleared after 72 h of administration. In the Kp56 infection model, the bacterial count significantly decreased in the blood and other organs by 4–7 log_10_ CFU/ml after 24 h of øKp_Pokalde_002 administration. Elimination half-life of øKp_Pokalde_002 was relatively shorter in the presence of host-bacteria Kp56 compared to phage only, suggesting rapid clearance of phage in the presence of susceptible host. Further, administration of the øKp_Pokalde_002 alone in healthy mice (*via* IP or oral) did not stimulate pro-inflammatory cytokines (TNF-α and IL-6). Also, treatment with øKp_Pokalde_002 resulted in a significant reduction of pro-inflammatory cytokines (TNF-α and IL-6) caused by bacterial infection, thereby reducing the tissue inflammation. In conclusion, the øKp_Pokalde_002 possess good PK/PD properties and can be considered as a potent therapeutic candidate for future phage therapy in carbapenem-resistant *K. pneumoniae* infections.

## Introduction

Antibiotic resistance has become one of the biggest challenges to the global public health. According to the World Health Organization (WHO), the world is heading towards a post-antibiotic era and it would force millions of people into extreme poverty and death by 2050 (WHO, 2017). The discovery of new class of antibiotics is often time consuming and requires tremendous investment, and as bacteria quickly become resistant to antibiotics, it will shortly be ineffective (Spellberg, 2014). As no new class of antibiotics has been discovered since the 1980s, researchers are warning about the imminent antibiotic resistance crisis of pandemic proportion if we fail to find effective alternative approaches to antibiotics in addition to development new classes of antibiotics. Recently, the ESKAPE (*Enterococcus faecium, Staphylococcus aureus, Klebsiella pneumoniae, Acinetobacter baumannii, Pseudomonas aeruginosa*, and *Enterobacter* species) pathogens are causing life-threatening infections throughout the world in both hospital and community settings with high morbidity and mortality (Paczosa and Mecsas, 2016). They are mostly multidrug-resistance (MDR) and acquire drug resistance potentially through different mechanisms such as drug inactivation, target modification, reduced permeability, or by increased efflux pump (Santajit and Indrawattana, 2016). Carbapenem-resistant *K. pneumoniae* is one of the ESKAPE pathogens categorized as critical by WHO, and research and development of new classes of antimicrobial agents is highly prioritized. A high prevalence of carbapenem-resistant *Enterobacteriaceae*, including *K. pneumoniae* infections, has also been reported in recent years in Southeast Asia including Nepal (Hsu et al., 2017; Nepal et al., 2017).

Bacteriophages (phages) are viruses that target specific bacterial species and has two distinct lifestyles: lytic and lysogenic, that dictate its role in bacterial biology. Recently, virulent phages (that strictly kill the host bacteria) have received heightened attention as a potent antimicrobial agent to treat bacterial infections, especially antibiotic resistant infections (Clokie et al., 2011). Phage therapy (using phage and its components as a therapeutic agent) has been known for more than 100 years and recently regained heightened interest as the modern understanding of phage biology, genetics, immunology, and pharmacology recognizes its use in mitigating the antibiotic resistance crisis (Young and Gill, 2015). Several studies have already demonstrated the safety and efficacy of phage therapy in systemic and tropical infections in both animal and human (Vinodkumar et al., 2008; Kumari et al., 2011; Pouillot et al., 2012; Furfaro et al., 2018; Wang et al., 2018). Phage therapy in humans is still routinely used in Georgia, Poland, and Russia, and Western countries like USA, UK, Belgium, France and Germany are using phages in therapeutics occasionally as personalized, magistral preparations and/or compassionate use to treat infections when all of the available antibiotics fail (Pirnay et al., 2018; Romero-Calle et al., 2019). Although there are more than 10 case reports published over last 10 years about phage therapy (Sybesma et al., 2018; Pirnay, 2020), and most of them showing encouraging results (Schooley et al., 2017; Dedrick et al., 2019; Petrovic Fabijan et al., 2020), it is yet to be adopted in mainstream medicine so far. Beside regulatory hurdles, one of the possible reasons for this is poor understanding of pharmacokinetics (PK) and pharmacodynamics (PD) of phages *in vivo*. Phages possess a unique tripartite dynamic relationship between their host bacteria and human immune system (Wahida et al., 2021) as they co-evolve and self-replicate within the human body in the presence of host bacteria (Payne and Jansen, 2003). As a result, the PK/PD of phages are distinct from those of classical antimicrobials. In addition, phages have ability to pass through body barriers, potentially eliciting an immune response (Barr et al., 2013; Dąbrowska and Abedon, 2019). It is necessary to understand the PK/PD of the phage in terms of biodistribution, bioavailability, clearance, and immune response *in vivo* (Caflisch et al., 2019). For successful phage therapy, route and dosage of phage administration must be assessed and standardized to each individual phage-bacteria combination (Payne and Jansen, 2003; Dąbrowska, 2019; Nilsson, 2019). In this study, we aimed to evaluate the PK/PD of a novel virulent (lytic) Klebsiella phage Kp_Pokalde_002 (GenBank ID: MT425185, hereafter referred as øKp_Pokalde_002) that infects carbapenem-resistant *K. pneumoniae* using oral and intraperitoneal (IP) route in a mouse model.

## Materials and Methods

### Ethical Clearance and Animal Model

Ethical approval was obtained for the use of animal prior to the study (Ethical approval No.161/2018) from Nepal Health Research Council (NHRC), Kathmandu. The protocol was also approved by the Ethical Review Board, NHRC. Female Swiss albino mice (6–8 weeks old) weighing 23 ± 2.5 g were purchased from Natural Products Research Laboratory (NPRL), Kathmandu. The animals were housed in an animal room at Central Department of Biotechnology, Tribhuvan University and fed with normal antibiotic-free diet. Chloroform vapor was used to anesthetize the mice and then euthanized by cervical dislocation before any invasive procedures. Each experiment was performed in triplicates.

### Bacterial Strain and Phage Amplification

A clinical isolate of *K. pneumoniae* (hereafter referred as Kp56) confirmed as a carbapenem-resistant strain (presence of gene *blaNDM1, blaKPC*) was obtained from the Microbiology Laboratory, Central Department of Biotechnology, Tribhuvan University (unpublished data). The bacteria were propagated in Luria-Bertani (LB) broth (HiMedia, India) at 37°C. A virulent øKp_Pokalde_002 (*Podoviridae*, C1 morphotype) isolated using Kp56 as a host was used in this study. The lytic-lifestyle and Gram-negative host of the phage was confirmed based on its physiochemical characteristics (Dhungana et al., 2021) and its genome analysis through PHACTS (https://edwards.sdsu.edu/PHACTS) (Mcnair et al., 2012).

The øKp_Pokalde_002 was amplified from glycerol stocks as described previously (Bourdin et al., 2014). Briefly, 1.0 ml overnight culture of the host bacteria (Kp56) was mixed with 100.0 ml LB broth and incubated at 37°C for 2.0 h with agitation (100 rpm) to reach an exponential growth phase (OD_600_ = 0.3). The phage stock, acclimatized to room temperature, was then added at a multiplicity of infection (MOI) of 10, and the culture was further incubated at 37°C in a shaking incubator (250 rpm) for 5.0 h until the media was visually clear. The phage lysate was centrifuged at 3220xg (Centrifuge 5810 R, Eppendorf, Hamburg, Germany) for 15 min at 4°C, and the supernatant was filtered through a 0.22 μm pore-size Whatman™ syringe filter (Sigma-Aldrich, Missouri, United States). The phage lysate was further purified and concentrated by isopycnic cesium-chloride (CsCl) density-gradient ultracentrifugation as described elsewhere (Sambrook and Russell, 2001).

### Phage/Bacteria Enumeration

Blood and homogenized tissue samples were serially diluted up to 10^-6^ in a 1.5 ml Eppendorf tubes. For bacterial count, 100 µ1 aliquot from each dilution was spread-plated on nutrient agar (NA) plates in duplicates and incubated at 37°C for 24 h. Similarly, for phage titer, the blood and homogenized tissue samples were centrifuged at 3220xg (Centrifuge 5810 R, Eppendorf, Hamburg, Germany) for 10 min at 4°C and filtered through a 0.22 µm pore size Whatman™ syringe filter (Sigma-Aldrich, Missouri, United States). The filtrate was serially diluted to up to 10^-8^ and phage titer was determined by Double Layer Agar (DLA) assay as described elsewhere. The phage and bacteria counts were corrected for tissue-fluid weights using following formula.

$$\frac{\#\text{ plaques or colonies}/\text{ml plated}\times \text{dilution factor}}{\#\text{ grams tissue}/\text{ml original homogenate}}=\text{PFU or CFU}/\text{gm of tissue}$$

### *In Vivo* Pharmacokinetics of øKp_Pokalde_002 Through Oral and IP Route

*In vivo* PK assessment was performed as described previously (Verma et al., 2009; Pouillot et al., 2012) with modifications. Seventy-two mice were divided into four groups [2 phage only and 2 vehicle (SM buffer) control, 18 mice in each group]. In a phage only control group, the first group of mice received 200 µl (1.2 x 10^8^ PFU/ml) of the highly purified øKp_Pokalde_002 *via* oral route while the same dosage of phage preparation was injected *via* IP route in the second group. The vehicle control group (third and fourth) received 200 µl of SM buffer only *via* oral and IP route, respectively. Three mice from each group were euthanized by cervical dislocation at 1 h, 4 h, 8 h, 24 h, 48 h, and 72 h after phage administration. Blood samples were collected in tubes containing 0.05 M EDTA anticoagulant by cardiac puncture. Tissue samples from lungs, liver, spleen, and kidneys were collected aseptically from euthanized mice and further divided into two parts. One part of each tissue was immersed in 10% formalin for histopathological examinations. Another part of tissue was weighed and homogenized in 1.0 ml PBS aseptically. The homogenized tissue was centrifuged at 10,000 rpm for 10 min at 4°C, and supernatant was filtered through a 0.22 µm pore size Whatman™ syringe filter (Sigma-Aldrich, Missouri, United States). The phage titer was determined by standard DLA technique as described elsewhere (Dhungana et al., 2021).

### *Klebsiella pneumoniae* Infection Model

In a separate study, 54 mice (3 groups, 18 in each group) were inoculated with 200 µl (1 x 10^8^ CFU/ml) of exponentially growing Kp56 intraperitonially. Immediately after bacterial inoculation, 200 µl of SM buffer was injected to all mice in the first group (sepsis control) and 200 µl of øKp_Pokalde_002 (1.2 x 10^8^ PFU/ml) was administered to all mice in second and third groups (treatment) through IP and oral routes, respectively. Three mice from each group were euthanized by cervical dislocation at 1 h, 4 h, 8 h, 24 h, 48 h, and 72 h post bacterial inoculation. Blood and tissue samples were collected and processed as described earlier to determine the phage titer and the levels of pro-inflammatory cytokines.

### Histology

Histological examination of the lung tissue was done as described previously (Singla et al., 2015) with modifications. Briefly, tissues were fixed with 10% formalin and embedded in paraffin wax. Serial sections of 4–6 µm thickness were cut using microtome, de-paraffinized, rehydrated, and stained with Hematoxylin and Eosin (H&E stain). The tissue sections were examined under the light microscope for histological changes.

### Cytokine Quantification

Pro-inflammatory cytokines: tumor necrosis factor alpha (TNF-α) and interleukin 6 (IL-6)] levels were measured in all Kp56 infected and øKp_Pokalde_002 treated mice. Total RNA was isolated from the blood samples using Direct-zol™ RNA MiniPrep Plus Kits (Zymo Research, USA), and cDNA was synthesized using iScript™ cDNA Synthesis Kit (Bio-Rad Laboratories, USA) following the manufacturer’s instruction. DNAse I (6 U/µl) was used to digest any residual DNA. Total RNA concentration was measured using NanoDrop 8000 (Thermo Fisher Scientific, USA) by spectrophotometric optical density measurement at 260/280 nm. The mRNA levels of TNF-α and IL-6 were measured by two-step relative qRT-PCR. The β-actin housekeeping gene was amplified as an internal control. Gene expressions were normalized to the expression of β-actin gene. The sequences of primers of IL-6, TNF-α, and β-actin are listed in **Supplementary Table S1**. The real time PCR was performed using SYBR^®^ Green Master Mix (2x) Kit in CFX Connect™ RT-PCR system (Bio-Rad Laboratories, USA). Melting curve analysis was performed after the amplification phase to eliminate the possibility of nonspecific amplification or primer-dimer formation. All samples were processed in duplicate, and the output level was reported as an average. The comparative CT method was used to calculate the relative expression ratio from the real time PCR efficiency and the CT (Livak and Schmittgen, 2001; Jain et al., 2006). mRNA expression level change was calculated using double delta Ct (DDCT) method, and the change in mRNA expression levels of cytokines was expressed as fold change.

$$\text{Fold change}=2^{-\Delta \Delta \text{Ct}}$$

where 2-ΔΔCt = [(Ct of gene of interest – Ct of internal control) sample A - (Ct of gene of interest – Ct of internal control) sample B].

### Data Interpretation and Statistical Analysis

Non-compartmental PK parameters: the peak plasma concentration (C_max_) and the time to reach peak plasma concentration (T_max_) were obtained by visual inspection of the data. The area under the plasma concentration-time curve (AUC) was calculated according to the linear trapezoidal rule up to the T_last_ phage concentration using GraphPad Prism 8 (Version 8.3.0). The half-life (T_1/2_) was calculated from the one-phase exponential regression equation (T_1/2_ = 0.693/K_el_) (Dufour et al., 2018; Chow et al., 2020). The elimination rate constant (K_el_) was estimated from the slope of the elimination phase of the log transformed plasma concentration-time curve fitted by the method of least squares. All elimination phase data with associated variability were included in the estimation. Data were expressed as mean ± standard error of mean (SEM). Comparisons of phage count and cytokine levels were performed by one-way ANOVA with Tukey’s multiple-comparison test and Student’s t-test. Inter mice PD variability was expressed as coefficient of variation (%CV). All statistical analysis were performed using GraphPad Prism 8 (Version 8.3.0), and differences with p < 0.05 were considered statistically significant.

## Results

### Pharmacokinetics

**Figure 1:**
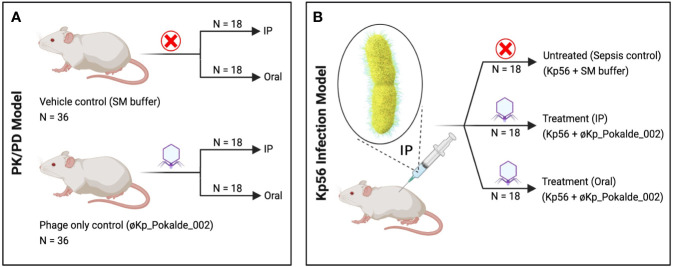
Schematic representation of the experimental design. **(A)** In PK/PD model, SM buffer (vehicle control) and same dose of purified øKp_Pokalde_002 (phage only control) was administered *via* both IP and oral route. **(B)** In Kp56 infection model, bacteria (*K. pneumoniae*) were administered *via* IP route only, while treatment (øKp_Pokalde_002) was administered *via* both IP and oral route. Figure created in BioRender.com. PK, pharmacokinetics; PD, pharmacodynamics; SM, Sodium Magnesium; IP, intraperitoneal.

We examined the PK/PD of øKp_Pokalde_002 administered through IP and oral routes in mice model in the presence and absence of host bacteria Kp56 (**Figure 1**). Mice that received only øKp_Pokalde_002 through IP or oral routes did not show any sign of illness during the experimental period (72 h post phage inoculation), and øKp_Pokalde_002 was detected in blood and other body tissues within the first hour of both IP and/or oral route of administrations.

**Figure 2:**
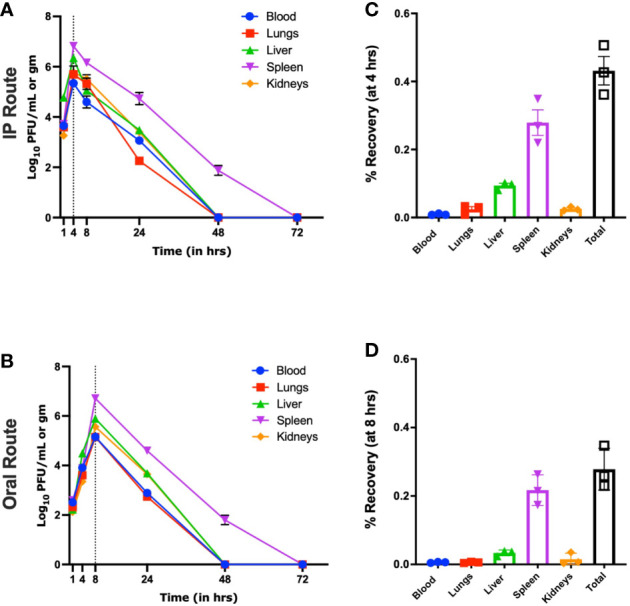
Pharmacokinetics of øKp_Pokalde_002 *in vivo via* IP and oral route in the absence of host bacteria Kp56. The phage concentration in log_10_ PFU/ml in blood, lungs, liver, spleen, and kidney after 1, 4, 8, 24, 48, and 72 h of phage administration *via* IP **(A)** and oral **(C)** route (200 µl of ~1 x 10^8^ PFU/ml). The result represents the mean from three independent experiments. Biodistribution of øKp_Pokalde_002 *via* IP **(B)** and oral **(D)** route at 4 h and 8 h, respectively. The dotted vertical line indicates T_max_. Percentage recovery was calculated by dividing phage titer at the respective time-point by the administered dose (n = 3 mouse per time point).

In an IP group and in the absence of host bacteria, maximum biodistribution of the øKp_Pokalde_002 was found at 4 h (43% of inoculated phage titer) post phage injection (**Figures 2A, B**). At 4 h, the phage titer was significantly higher in spleen (6.8 ± 0.10 log_10_ PFU/ml, 6.69 x 10^7^ PFU/ml) compared to blood (5.3 ± 0.12 log_10_ PFU/ml, 2.22 x 10^5^ PFU/ml), lungs (5.6 ± 0.4 log_10_ PFU/ml, 5.78 x 10^5^ PFU/ml), liver (6.3 ± 0.05 log_10_ PFU/ml, 2.25 x 10^6^ PFU/ml), and kidneys (5.8 ± 0.10 log_10_ PFU/ml, 6.04 x 10^5^ PFU/ml) (p < 0.0001, two-way ANOVA with Tukey’s multiple comparisons) (**Figure 2A** and **Supplementary Table S2**). After 4 h, there was a gradual decrease in phage titer in all organs and the phage was completely cleared within 48 h of phage inoculation except from spleen, where the complete clearance was seen at 72 h.

Similarly, in an oral route and in the absence of the host bacteria, maximum biodistribution of the øKp_Pokalde_002 was found at 8 h (28%) post phage administration (**Figures 2C, D**). At 8 h, the phage titer was significantly higher (p < 0.0001, two-way ANOVA with Tukey’s multiple comparisons test) in spleen (6.7 ± 0.09 log_10_ PFU/ml, 5.21 x 10^6^ PFU/ml) compared to blood (4.8 ± 0.1 log_10_ PFU/ml, 1.45 x 10^5^ PFU/ml), lungs (5.1 ± 0.13 log_10_ PFU/ml, 1.44 x 10^5^ PFU/ml), liver (5.9 ± 0.12 log_10_ PFU/ml, 8.10 x 10^5^ PFU/ml), and kidneys (5.5 ± 0.35 log_10_ PFU/ml, 4.50 x 10^5^ PFU/ml) (**Figure 2C**). After 8 h, the phage titer gradually decreased and completely cleared from all organs within 48 h of phage administration except spleen, where the complete clearance was seen at 72 h. As expected, we further observed that relative bioavailability was lower when phage was administered through oral route compared to IP (**Table 1**) in the absence of host bacteria Kp56. Although the results were similar in the presence of host Kp56, the relative bioavailability of phage was higher in blood and spleen when administered orally compared to IP.

**Table 1:** Estimated pharmacokinetic parameters of virulent phage (øKp_Pokalde_002) in the absence and in the presence of host *K. pneumoniae* (Kp56).

Organ	Blood		Lungs		Liver		Spleen		Kidneys		
Route of administration	IP	Oral	IP	Oral	IP	Oral	IP	Oral	IP		Oral
**Parameters**	**In the absence of host bacteria (Kp56)** **Administered dose: 200 µl of 1.2 × 10^8^ PFU/ml of øKp_Pokalde_002**										
**C_max_ (pfu/ml)**	222778	72311	578611	14471	2258318	87056	6694839	521210	604444	45097	
**T_max_ (h)**	4	8	4	8	4	8	4	8	4	8	
**Vd (L)**	1.27	9.7	0.86	18.8	0.16	4.3	0.07	2.07	0.60	12.8	
**T_1/2_ (h)**	8.29	8.35	7.21	8.45	7.34	7.13	6.87	6.58	7.34	7.49	
**CL (L/h)**	0.21	1.32	0.15	2.36	0.03	0.62	0.01	0.3	0.1	1.6	
**AUC_0-t_ (pfu/h/ml)**	269539	155155	807450	149419	2407478	848459	8248503	5262198	948204	458160	
**Relative bioavailability (F)**	58%		19%		35%		64%		48%		
	**In the presence of host bacteria (Kp56)** **Administered dose: 200 µl of 1.0 × 10^8^ CFU/ml of Kp56 + 200 µl of 1.2 × 10^8^ PFU/ml of øKp_Pokalde_002**										
**C_max_ (pfu/ml)**	2923000	3315027	34693333	2107333	56589196	16643667	293940000	579333333	23068000		1695000
**T_max_ (h)**	8	24	8	24	24	24	24	24	24		24
**Vd (L)**	0.15	0.48	0.01	0.2	0.005	0.009	0.01	0.02	0.006		0.03
**T_1/2_ (h)**	7.51	5.24	6.26	5.37	5.85	4.94	6.87	5.46	5.20		5.44
**CL (L/h)**	0.02	0.06	0.002	0.02	0.0009	0.001	0.001	0.002	0.001		0.005
**AUC_0-t_ (pfu/h/ml)**	3263704	4075882	43003899	2979558	97074444	25790850	399112587	626186433	190270651		3977100
**Relative bioavailability (F)**	125%		7%		27%		157%		2%		

C_max_, maximum observed plasma concentration; T_max_, time to the C_max_; V_d_, Volume of distribution; T_1/2_, elimination half-time; CL, clearance; AUC_0‒t_, area under the concentration-time curve from time 1 h to the last quantifiable concentration. Relative bioavailability (F) was calculated using the following formula: F, AUC_0‒t_ (oral)/AUC_0‒t_ (IP)×100%.

**Figure 3:**
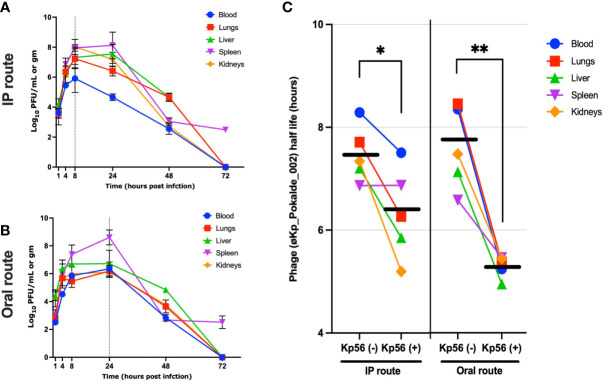
Pharmacokinetics of øKp_Pokalde_002 *in vivo* and half-life of øKp_Pokalde_002 in the presence and absence of host bacteria Kp56 in mice when administered *via* IP and oral routes. The phage concentration in log_10_ PFU/ml in blood, lungs, liver, spleen, and kidneys after 1, 4, 8, 24, 48, and 72 h in Kp56 treatment group after administration of phage *via* IP **(A)** and oral **(B)** route (200 µl of ~1 x 10^8^ PFU/ml). The dotted vertical line indicates T_max_. **(C)** The overall elimination half-life of øKp_Pokalde_002 is lower when host bacteria are present, signifying rapid clearance of phage from circulation in the presence of susceptible host. The individual data point represents an average from three replicates from three mouse. The horizontal line represents the grand mean.

In the presence of host bacteria Kp56, maximum titer of the øKp_Pokalde_002 was found at 8 h post phage injection (IP) and 24 h (oral) (**Figures 3A, B**) and gradually decreased after 24 h. In both group, maximum phage titer was found in the spleen at 24 h post phage injection. However, in contrast to phage inoculations without host, the phage did not clear from spleen until 72 h when inoculated with host Kp56.

We further report that mean elimination half-lives of øKp_Pokalde_002 in different organs were route independent [mean = 7.48 h, CV = 7.2% (IP) and mean = 7.6 h, CV = 10.5% (oral)] but the half-life was significantly lower [mean = 6.33 h, CV = 14.0% (IP) and mean = 5.3 h, CV = 4.0% (oral)] when susceptible host (Kp56) was present (**Table 1**) *via* both IP (p = 0.03, r^2^ = 0.72, paired t-test) and oral (p = 0.0034, r^2^ = 0.90, paired t-test) (**Figure 3C**) route possibly because of strong immune response from mice against bacteria and phage in the presence of Kp56 suggesting rapid clearance.

### Pharmacodynamics

**Figure 4:**
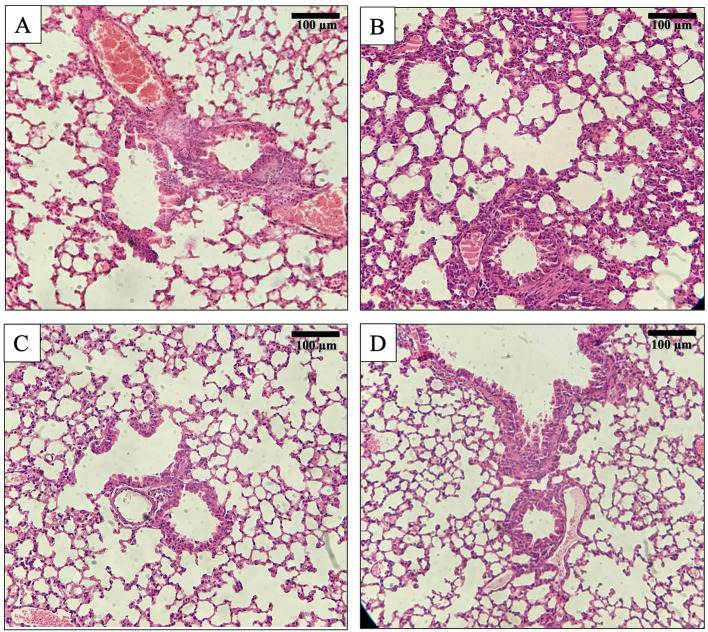
Histology of mouse lung tissue sections after Hematoxylin and Eosin (H&E) staining at 200x magnification. **(A)** Lungs’ tissue of a normal mouse. **(B)** Lungs’ tissue of bacteria *K pneumoniae* (Kp56) infected mouse showing interstitial infiltration by neutrophils and macrophages with rupture of alveoli. **(C)** Lungs’ tissue of mouse treated with øKp_Pokalde_002 *via* IP route. **(D)** Lungs’ tissue of mouse treated with øKp_Pokalde_002 *via* oral route.

The groups of mice in PK/PD model (not infected by Kp56) that received øKp_Pokalde_002 *via* IP or oral route showed only mild to moderate alveolar wall thickening and remarkably reduced neutrophil infiltration in perivascular and peri bronchial areas (**Figure 4**). Moreover, they also did not show any significant histological changes compared to the vehicle control (SM buffer only) group at 24 h post phage inoculation. On the other hand, in the Kp56 infection model, bacterial count increased exponentially in the blood and lungs for up to 24 h when treated with SM buffer only (untreated group), while the bacterial count gradually decreased after 8 h when treated with øKp_Pokalde_002 (treatment group) *via* both IP and oral routes. The bacterial count significantly reduced by 4–7 log_10_ CFU/ml in the blood (p < 0.001) and lungs (p < 0.05) at 24 h of øKp_Pokalde_002 administration compared to untreated (Kp56 + SM buffer) group (**Supplementary Figure S3**, two-way ANOVA with Tukey’s multiple comparisons). Further, comparison of histological changes in the lung tissues from untreated group (Kp56 + SM buffer) and treatment group (Kp56 + øKp_Pokalde_002) revealed a noticeable interstitial infiltration by neutrophils and macrophages with severe thickening, congestion, and destruction of alveolar wall in the lungs of untreated group. Meanwhile, orally treated group showed relatively increased neutrophil infiltration in the alveoli (lung tissues) compared to the IP-treated group.

**Figure 5:**
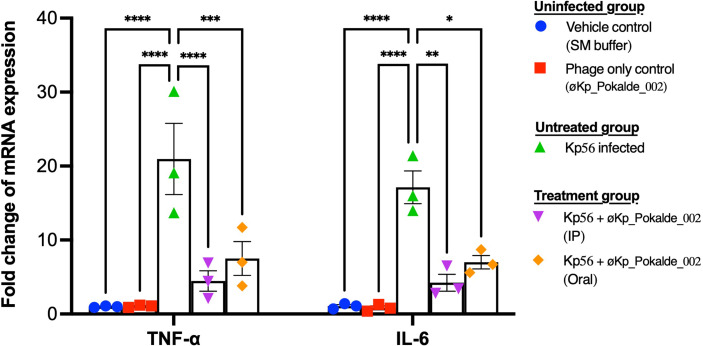
Pro-inflammatory cytokine TNF-α and IL-6 levels in the plasma of mice (24 h post infection). Both TNF-α and IL-6 mRNA levels were significantly higher in Kp56 infected mice compared to uninfected and treated mice (p < 0.05) *via* both IP and oral routes. There was negligible fold increment of TNF-α and IL-6 mRNA level in vehicle control (SM buffer) and phage only control (øKp_Pokalde_002). Levels of TNF-α and IL-6 mRNA were normalized to β-actin mRNA levels and were expressed as n-fold (2^-∆∆Ct^) increase with reference to the control groups. Results are shown as means ± SEM from triplicate experiments. The y-axis values represent the fold changes of mRNA relative to the β-actin mRNA in the same sample. The statistical comparison was done by two-way ANOVA. *p < 0.05, **p < 0.01, ***p < 0.001, ****p < 0.0001.

The expression level of two pro-inflammatory cytokine (TNF-α and IL-6) in blood was analyzed to evaluate the tissue inflammation either by øKp_Pokalde_002 or by Kp56. Cytokine expression levels in the control group (SM buffer only), phage administered group (øKp_Pokalde_002 only), Kp56 infected group (Kp56 + SM buffer), and phage-treated groups (Kp56 + øKp_Pokalde_002) were compared. A significant upregulation of both pro-inflammatory cytokines’ TNF-α and IL-6 (p < 0.0001, Tukey’s multiple comparisons test) was observed in the Kp56 infected (Kp56 + SM buffer) group compared to the control (SM buffer only) group, and at 24 h post infection, the increment in the TNF-α and IL-6 was 21.0-fold and 17.1-fold, respectively. Changes in TNF-α and IL-6 in phage-only administered group were 1.1-fold and 0.9-fold, respectively, compared to vehicle control (SM buffer only) arm. Interestingly, the levels of cytokine expressions in the phage-treated groups *via* both IP and oral route were significantly lower compared to Kp56 infected (Kp56 + SM buffer, untreated) arm (p < 0.05, Tukey’s multiple comparisons test). The fold changes in cytokine TNF-α and IL-6 expression levels in phage-treated (Kp56 + øKp_Pokalde_002) groups compared to the uninfected control (phage only) arm are depicted in **Figure 5**.

## Discussion

Phage therapy is considered one of the promising alternatives to treat infections caused by MDR bacteria (Romero-Calle et al., 2019). PK/PD are fundamental parameters for better understanding the success of phage therapy and obtaining regulatory approval (Dąbrowska and Abedon, 2019). In this study, we focused on PK/PD of a novel øKp_Pokalde_002 that infects carbapenem-resistant *K. pneumoniae* using oral and IP routes of administration in a mouse model. Our results showed that øKp_Pokalde_002 rapidly distributed into the systemic circulation within an hour of administration *via* both oral and IP route. A relatively higher concentration of øKp_Pokalde_002 was recovered from plasma while injecting the phage through IP route compared to oral administration. When phage was administrated in mice through the IP route, highest phage titer in the blood reached after 4 h post administration, significantly decreased after 8 h, and negligible count was observed after 24 h. The result suggests that the phage net phage elimination is observed after 4 h if injected intraperitonially in the absence of host bacteria. The result is consistent with other studies where it is reported that the phages take 2–4 h to reach its maximum count in blood and is subsequently decreased after 12 h (Bogovazova et al., 1992; Capparelli et al., 2006; Kumari et al., 2010; Tiwari et al., 2011). Further, recovery of phages from blood and other tissue after oral administration shows that øKp_Pokalde_002 survived the gut environment and crossed the gut barrier to reach systemic circulation in mice subsequently reaching to different organs which is consistent with reports from other researchers (Cerveny et al., 2002; Gorski and Weber-Dabrowska, 2005). Several mechanisms have been proposed for phage absorption in the gastrointestinal tracts such as intestinal permeability and intestinal transport. Although the mechanism of controlling viral translocation remains unknown, researchers suggested that the phage passage is determined by various factors, including stomach acidity, phage concentration, and interactions with gut immune cells. Micropinocytosis may be a major endocytic pathway to translocate the phage from the intestinal wall into systemic circulation (Dąbrowska, 2019).

In our experiment, phages were recovered from blood, lungs, liver, and kidneys for up to 24 h and for up to 48 h in the spleen in the absence of host bacteria *via* both IP and oral route. However, there was significant difference in phage distribution, bioavailability, and elimination between IP and oral routes of administration. øKp_Pokalde_002 reached its maximum titer in blood at 4 h (2.3 x 10^5^ PFU/ml) when administered through IP route which was relatively higher compared to administration *via* oral route (4.04 x 10^3^ PFU/ml). Similar findings have been reported previously (Keller and Engley, 1958; Cerveny et al., 2002; Oliveira et al., 2009; Jun et al., 2014). Additionally, overall relative bioavailability of øKp_Pokalde_002 when administered *via* oral route (at 8 h) was lower compared to IP route (at 4 h) in both the absence and/or presence of host bacteria. The reason for reduced bioavailability *via* oral route compared to IP might be due to slow absorption of the phage in the gastrointestinal tract to reach into the systemic circulation. However, it must be noted that because of the low sampling resolution, the T_max_ could be higher than 4 h and 8 h in IP and oral administration respectively. As øKp_Pokalde_002 was stable within wide pH range (3–11) with minimal decrease in phage titer and did not show significant inactivation at 25°C and 37°C (Dhungana et al., 2021), the phage was well tolerated in mice gut with low acidity, making it a good candidate for oral phage therapy. It therefore appears that the øKp_Pokalde_002 is relatively stable in the mouse body when administered *via* the oral route but their availability is comparatively lower and slower. Similar findings have also been reported by Otero et al. (2019) and were able to recover orally administered encapsulated as well as non-encapsulated phages from various organs. Further, the inter mice PD variability [coefficient of variation (%CV)] was more pronounced in oral (7–78%) compared to IP (5–56%) route (**Supplementary Table S3**). The inter mice variability was profound in groups of Kp56 infection model. In addition to differential absorption of øKp_Pokalde_002 between animals and innate immunity, the higher variability between mice in the oral group may be because of the inconsistent neutralization of phages in the gut environment caused by gut acidity (feeding habit of mice). The phage absorption in the gastrointestinal tract is affected by various factors like gut acidity and gut permeability and is thus relatively slow. As such, lower phage particles reach into the blood stream through oral route compared to the IP route, which makes clinical application of phage *via* oral route for systemic infection unfavorable (Wolochow et al., 1966).

Further, the results suggest that liver and spleen are the most common organs of phage accumulation, suggesting phages are cleared by organs of the reticuloendothelial system such as the spleen, liver, and other filtering organs (Merril et al., 1996; Dąbrowska and Abedon, 2019). Similar results of non-homogenous biodistribution and preferential accumulation of phages in organs like spleen and liver has also been observed in anti-pseudomonal phage in mice (Lin et al., 2020) and rabbit *in vivo* models (Uhr and Weissman, 1965). Further, phages are also reported in urine of human (Hildebrand and Wolochow, 1962) and animal models like rats (Wolochow et al., 1966) and rabbits (Schultz and Neva, 1965) after systemic injection which supports our finding that phage can pass through the renal filter. The role of the kidneys in the clearance of phages has also been observed in fish, where phages were detected in fish kidney a month after phage administration (Russell et al., 1976).

The PK of phages are fundamentally different from those of chemical drugs due to the self-replicative nature of phages in the presence of susceptible bacteria, its absorption rate, and clearance by host’s immunity (Dąbrowska, 2019); thus, phage half-life cannot be estimated by conventional approach. Although researchers have demonstrated prolonged phage half-life *in vivo* with encapsulation of phage (Colom et al., 2015; Singla et al., 2016), the half-life of phage in the presence of a host is scarce. Using one phase decay model, our study showed that there was no significant difference in elimination half-life of øKp_Pokalde_002 when administered *via* IP and oral routes suggesting phage half-life to be route independent. However, the phage had a shorter elimination half-life in the blood and other organs when Kp56 was present, although phage titer was relatively higher in treatment groups compared to phage only control groups. This clearly suggests that phages can exponentially increase their number *in vivo* infecting and lysing the susceptible host bacteria and is cleared more rapidly by strong immune response developed against host bacteria (nonspecific) and phage itself (anti-phage). This may explain why multiple injections of phage is required for phage therapy, although theoretically phages are self-multiplying. However, a study on Klebsiella phage by Soleimani Sasani and Eftekhar (2020) found half-life in blood (4 h) when phages were administered intraperitoneally [100 µl of 10^10^ PFU/ml (*Myoviridae*)] and 8 h in lungs, whereas Kumari et al. (2010) reported maximum recovery from blood, peritoneal fluid, lungs, and skin at 6 h post IP injection [250 µl of 10^10^ PFU/ml (*Podoviridae*)]. Moreover, the half-life of phage seems to be comparable to that of antibiotics in animal models (Chang et al., 1991; Griffith et al., 2003) which ranges from 0.5 h to more than 7 h which makes it a good drug candidate against bacterial infections. However, more research is required in *in vivo* models to understand the half-life of different phages in the presence of susceptible host as this is important in designing the therapeutic dose of phage.

The histology results also revealed that the lung tissue of the øKp_Pokalde_002 administrated mice had a similar histological picture with reference to the wild-type and SM buffer only administrated mice group. Similar results of no detrimental histological effects were also observed by Gangwar et al. (2021) in various organs of Charles Foster rats when challenged by high (10^15^ and 10^20^ PFU/ml) of Klebsiella phage orally. Pro-inflammatory cytokines, TNF-α and IL-6, are useful markers of infection severity  (Bozza et al., 2007). Present study revealed that there was negligible upregulation of pro-inflammatory cytokines (TNF-α, and IL-6) with the øKp_Pokalde_002 administrated *via* both IP and oral routes. In contrast, there was significant upregulation of the cytokines in the mice infected with the Kp56. Upon infection, pro-inflammatory cytokines are released by the macrophages to adhere the other inflammatory cells at the infection site (Liu et al., 2016). The expression of the cytokines was dropped after 24 h of the øKp_Pokalde_002 administration in both IP and oral routes signifying removal of Kp56. The result supports the findings of other researchers who have reported significant reduction in cytokines levels in phage-treated mice (Watanabe et al., 2007; Wang et al., 2016). Phage lysates that are prepared from the gram-negative bacteria may contain bacterial endotoxins. Endotoxins are highly immunogenic, which could trigger the inflammatory response. An overexpression of cytokines leads to a septic shock and consecutive death (Cavaillon, 2018). Phage preparation should be necessarily purified to ensure the low level of the endotoxin and other bacterial contamination. However, in our study, we did not measure the level of endotoxin in the phage lysate. Although researchers have highlighted that phage therapy causes lysis of the host bacteria within the body, thus releasing endotoxins/enterotoxins, which may induces higher levels of TNF-α and IL-6 causing septic shock (Hagens et al., 2004), the øKp_Pokalde_002 did not induce a significant inflammatory response in mice indicating a good PD efficiency. However, Chow et al. (2020) also reported that such upregulation of pro-inflammatory cytokines was transient and was diminished over time. Our results suggested that systemic inflammation of the tissues is lower in phage-treated mice as compared to the untreated. The histological findings of the lung tissue also support these findings.

In conclusion, PK/PD of øKp_Pokalde_002 *in vivo* were assessed. Inflammatory response, half-life, and biodistribution of the phage in blood, lungs, liver, kidneys, and spleen of mouse model were determined at different time interval *via* IP and oral routes of phage administration. The øKp_Pokalde_002 distributed more rapidly into the systemic circulation *via* the IP route compared to oral route. Importantly, the øKp_Pokalde_002 did not elicit any notable inflammation in lung tissues. Further, treatment by øKp_Pokalde_002 significantly reduced the inflammations caused by bacterial infection and downregulated the levels of the pro-inflammatory cytokine (TNF-α and IL-6) expression.

To the best of our knowledge, this is the first study that evaluates the PK/PD of a virulent Klebsiella phage that infects carbapenem-resistant clinical isolate of *K. pneumoniae via* IP and oral routes of administration. However, more work is necessary to better understand the PK/PD of the phage using different dose regimes and time of the phage exposure in *in vivo* model.

## Data Availability Statement

The original results of the study are included in the article. Further inquiries can be directed to the corresponding author.

## Ethics Statement

Ethical approval was obtained for the use of animal prior to the study from Nepal Health Research Council (NHRC), Nepal (Ethical approval No.161/2018). The protocol was also approved by the Ethical Review Board, NHRC.

## Author Contributions

GD and RM conceived the idea and designed the study. GD and MR performed the experiments. GD, MR, and RN analyzed the data. GD and RN drafted the manuscript. RM supervised the project. All authors contributed to the article and approved the submitted version.

## Funding

This work was partially supported by the ‘PhD Fellowship and Research Support’ awarded to GD by the University Grants Commission, Nepal (UGC-Nepal) (Award Numbers: PhD/73-74/S and T-07).

## Conflict of Interest

The authors declare that the research was conducted in the absence of any commercial or financial relationships that could be construed as a potential conflict of interest.

## Publisher’s Note

All claims expressed in this article are solely those of the authors and do not necessarily represent those of their affiliated organizations, or those of the publisher, the editors and the reviewers. Any product that may be evaluated in this article, or claim that may be made by its manufacturer, is not guaranteed or endorsed by the publisher.

## Supplementary Material

Supplementary Figure 1 Analysis of øKp_Pokalde_002 genome for prediction of its lifestyle and host. The lytic-lifestyle and Gram-negative host of the phage was confirmed based on its physiochemical characters (Dhungana et al., 2021) and analysis of its amino acid sequences through PHACTS (https://edwards.sdsu.edu/PHACTS/index.php).

Supplementary Figure 2 Area under the curve (AUC) from all groups of mice. **(A)** Phage pharmacokinetics and AUC after administration of phage *via* IP route in the absence of host Kp56. **(B)** Phage pharmacokinetics and AUC after administration of phage *via* oral route in the absence of host Kp56. **(C)** Phage pharmacokinetics and AUC after administration of phage *via* IP route in the presence of host Kp56. **(D)** Phage pharmacokinetics and AUC after administration of phage *via* oral route in the presence of host Kp56.

Supplementary Figure 3 Quantification of bacterial burden in lungs and blood of mice from different group. Bacterial load was significantly decreased in phage treated group compared to group that only received SM buffer as treatment control. The bacterial burden in lungs **(A)** was similar to the bacterial burden in blood of phage treated group. Although the burden of bacterial significantly decreased, it appears that oral treatment is relatively less effective compared to IP and IP-one hour delay. The color-dotted line indicates the non-linear exponential growth fit (log population).

Supplementary Table 1 Primers used in the study.

Supplementary Table 2 P-values at 4 h (IP administration) and 8 h (oral administration) of øKp_Pokalde_002 in the absence of host bacteria Kp56.
