# Supplementary figures and images for: Pharmacokinetics and Pharmacodynamics of a Novel Virulent Klebsiella Phage Kp_Pokalde_002 in a Mouse Model
Source: Front Cell Infect Microbiol. 2021 Aug 16;11:684704. doi: 10.3389/fcimb.2021.684704 (PMC8415502; doi:10.3389/fcimb.2021.684704)

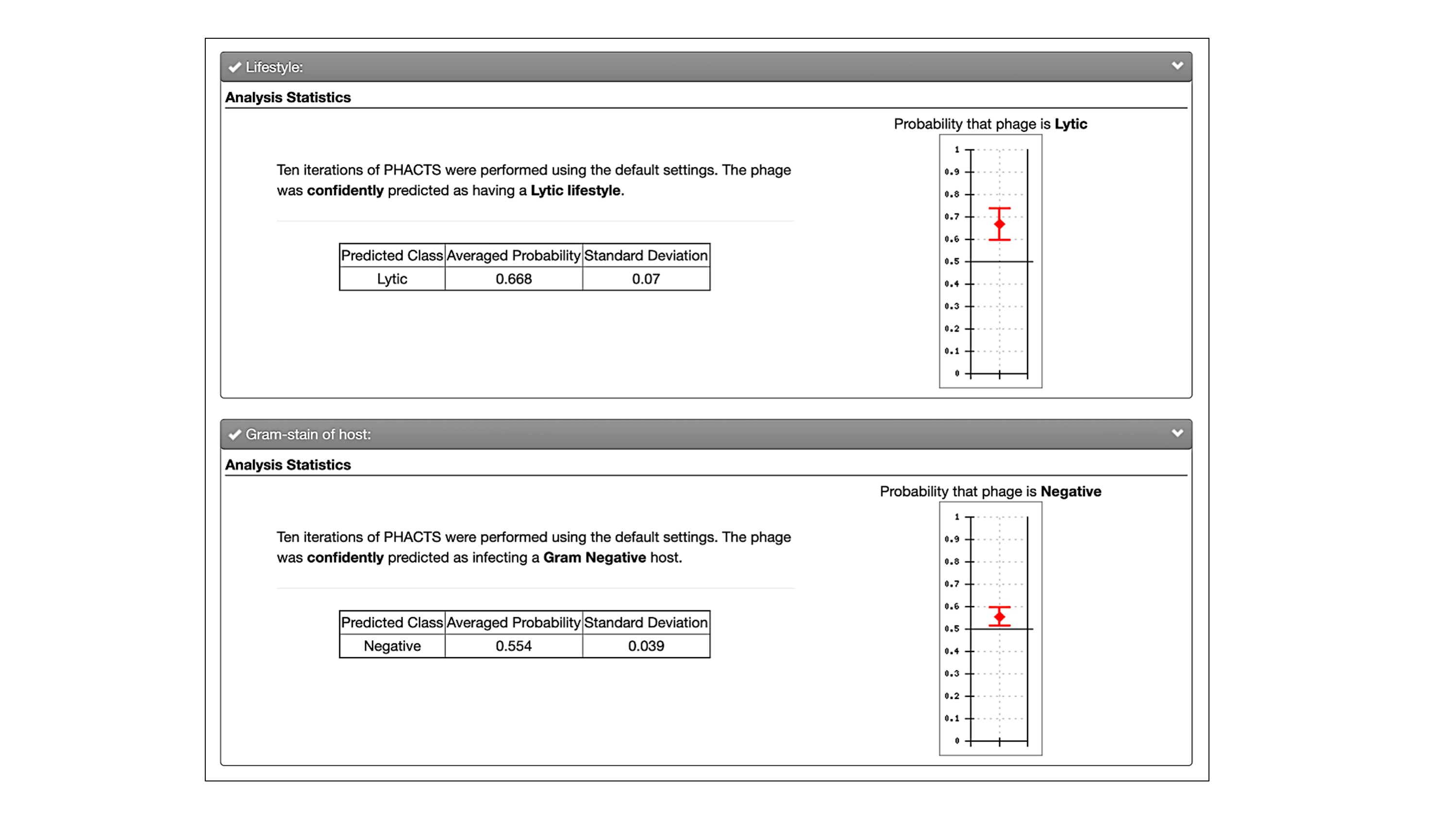

Supplement: Supplementary Figure 1 — Analysis of øKp_Pokalde_002 genome for prediction of its lifestyle and host. The lytic-lifestyle and Gram-negative host of the phage was confirmed based on its physiochemical characters (Dhungana et al., 2021) and analysis of its amino acid sequences through PHACTS (https://edwards.sdsu.edu/PHACTS/index.php). [file Image_1.jpeg]

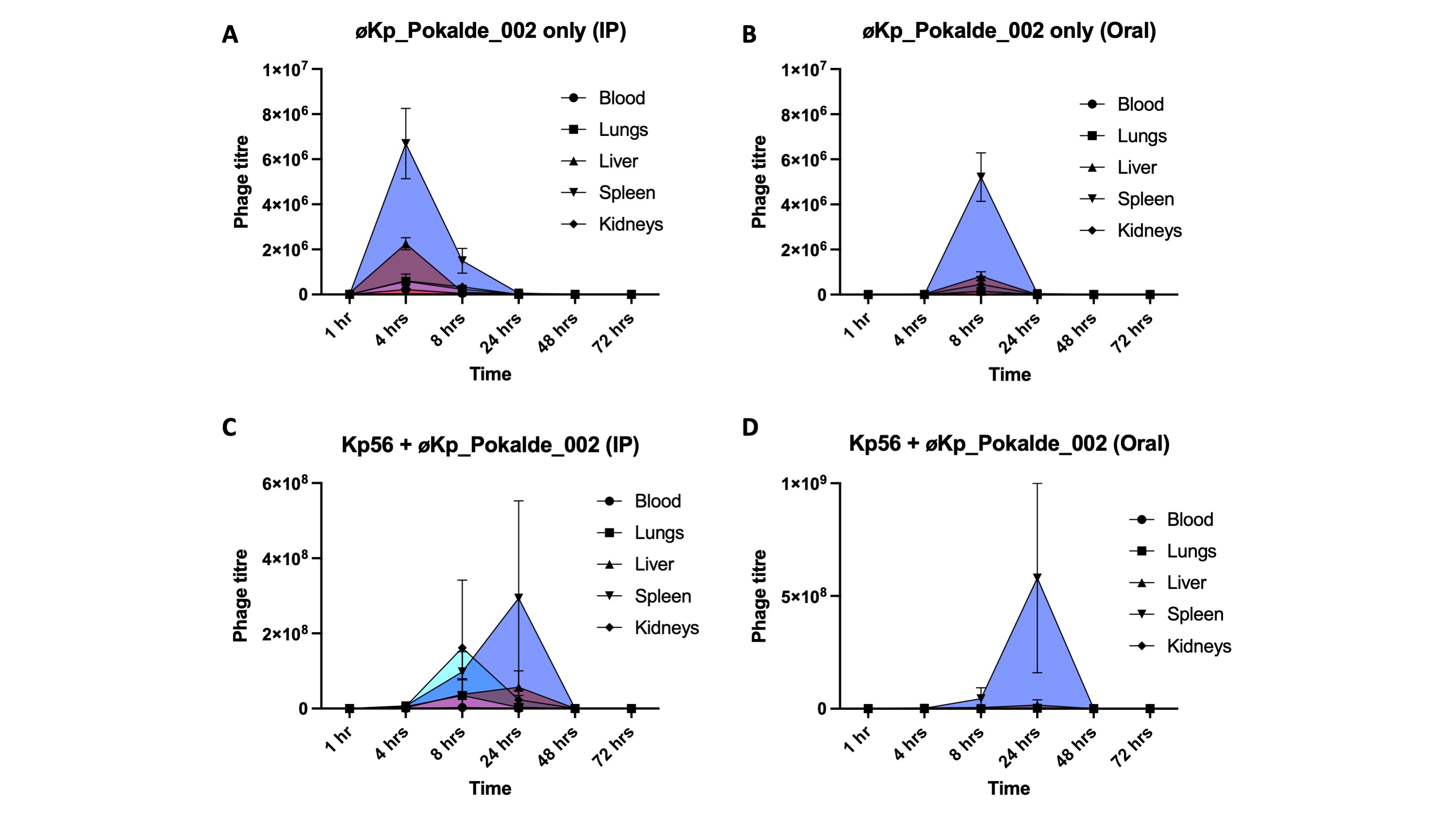

Supplement: Supplementary Figure 2 — Area under the curve (AUC) from all groups of mice. (A) Phage pharmacokinetics and AUC after administration of phage via IP route in the absence of host Kp56. (B) Phage pharmacokinetics and AUC after administration of phage via oral route in the absence of host Kp56. (C) Phage pharmacokinetics and AUC after administration of phage via IP route in the presence of host Kp56. (D) Phage pharmacokinetics and AUC after administration of phage via oral route in the presence of host Kp56. [file Image_2.jpeg]

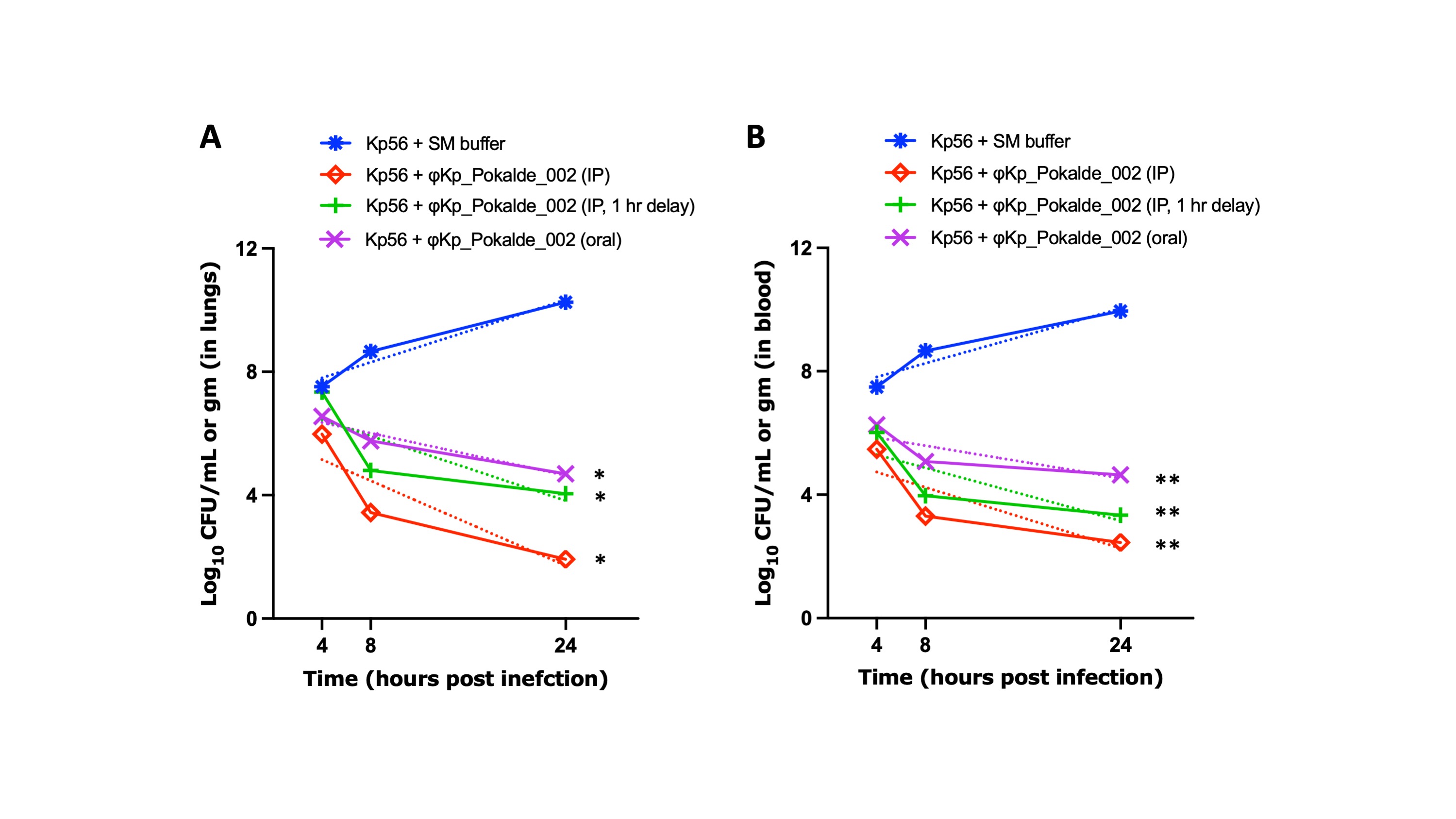

Supplement: Supplementary Figure 3 — Quantification of bacterial burden in lungs and blood of mice from different group. Bacterial load was significantly decreased in phage treated group compared to group that only received SM buffer as treatment control. The bacterial burden in lungs (A) was similar to the bacterial burden in blood of phage treated group. Although the burden of bacterial significantly decreased, it appears that oral treatment is relatively less effective compared to IP and IP-one hour delay. The color-dotted line indicates the non-linear exponential growth fit (log population). [file Image_3.jpeg]
