# Supplementary material for: Pharmacokinetics and Pharmacodynamics of a Novel Virulent Klebsiella Phage Kp_Pokalde_002 in a Mouse Model
Source: Front Cell Infect Microbiol. 2021 Aug 16;11:684704. doi: 10.3389/fcimb.2021.684704 (PMC8415502; doi:10.3389/fcimb.2021.684704)
Supplement: Supplementary Table 1 — Primers used in the study. [file Table_1.docx]

**Supplementary Table S1**

| Table S1 \| Primers used in the study. | |
| --- | --- |
| Cytokine | **Primer sequences** |
| IL-6 Forward | 5′-GAGGATACCACTCCCAACAGACC-3′ |
| IL-6 Reverse | 5′-AAGTGCATCATCGTTGTTCATACA-3′ |
| TNF-α Forward | 5′-CATCTTCTCAAAATTCGAGTGACAA-3′ |
| TNF-α Reverse | 5′-TGGGAGTAGACAAGGTACAACCC-3′ |
| β-actin Forward | 5’-CTGTCCCTGTATGCCTCTG-3’ |
| β-actin Reverse | 5’-ATGTCACGCACGATTTCC-3’ |
