# Supplementary material for: Pharmacokinetics and Pharmacodynamics of a Novel Virulent Klebsiella Phage Kp_Pokalde_002 in a Mouse Model
Source: Front Cell Infect Microbiol. 2021 Aug 16;11:684704. doi: 10.3389/fcimb.2021.684704 (PMC8415502; doi:10.3389/fcimb.2021.684704)
Supplement: Supplementary Table 2 — P-values at 4 h (IP administration) and 8 h (oral administration) of øKp_Pokalde_002 in the absence of host bacteria Kp56. [file Table_2.docx]

**Supplementary Table S2**

| Table S2 \| P-values at 4 hr (IP administration) and 8 hrs (oral administration) of  øKp_Pokalde_002 in absence of host bacteria Kp56. | | | | |
| --- | --- | --- | --- | --- |
|  | **4 hrs (IP): Phage only** | | **8 hrs (Oral): Phage only** | |
| Blood vs. Lungs | ns | 0.6401 | ns | >0.9999 |
| Blood vs. Liver | **** | <0.0001 | ** | 0.0024 |
| Blood vs. Spleen | **** | <0.0001 | **** | <0.0001 |
| Blood vs. Kidneys | ns | 0.5763 | ns | 0.3968 |
| Lungs vs. Liver | **** | <0.0001 | ** | 0.0024 |
| Lungs vs. Spleen | **** | <0.0001 | **** | <0.0001 |
| Lungs vs. Kidneys | ns | >0.9999 | ns | 0.3926 |
| Liver vs. Spleen | **** | <0.0001 | **** | <0.0001 |
| Liver vs. Kidneys | **** | <0.0001 | ns | 0.2366 |
| Spleen vs. Kidneys | **** | <0.0001 | **** | <0.0001 |
