# Supplementary material for: Pharmacokinetics and Pharmacodynamics of a Novel Virulent Klebsiella Phage Kp_Pokalde_002 in a Mouse Model
Source: Front Cell Infect Microbiol. 2021 Aug 16;11:684704. doi: 10.3389/fcimb.2021.684704 (PMC8415502; doi:10.3389/fcimb.2021.684704)
Supplement: Supplementary file 6 [file Table_3.docx]

**Supplementary Table S3**

| Table S3 \| Inter mice variability (%CV) between IP and oral groups of mice. | | | | | | | | | | |
| --- | --- | --- | --- | --- | --- | --- | --- | --- | --- | --- |
| Time | **%CV (Blood)** | | **%CV (Lungs)** | | **%CV (Liver)** | | **%CV (Spleen)** | | **%CV (Kidneys)** | |
|  | **IP** | **Oral** | **IP** | **Oral** | **IP** | **Oral** | **IP** | **Oral** | **IP** | **Oral** |
| In absence of host bacteria (Kp56)  Administered dose: 200 µl of 1.2×10^8^ PFU/mL of øKp_Pokalde_002 | | | | | | | | | | |
| 1 hour | 15.19 | 27.86 | 5.90 | 37.32 | 23.90 | 14.94 | 20.20 | 7.18 | 8.40 | 19.83 |
| 4 hours | 23.60 | 18.75 | 56.59 | 13.61 | 12.17 | 38.10 | 23.34 | 20.44 | 22.69 | 36.29 |
| 8 hours | 48.48 | 23.49 | 43.12 | 30.07 | 17.93 | 25.01 | 36.97 | 20.64 | 33.71 | 78.44 |
| 24 hours | 5.24 | 11.16 | 24.34 | 12.78 | 14.88 | 20.41 | 45.26 | 43.06 | 16.03 | 40.82 |
| 48 hours | 0.00 | 0.00 | 0.00 | 0.00 | 0.00 | 0.00 | 46.07 | 73.35 | 0.00 | 0.00 |
| 72 hours | 0.00 | 0.00 | 0.00 | 0.00 | 0.00 | 0.00 | 0.00 | 0.00 | 0.00 | 0.00 |
| In presence of host bacteria (Kp56)  Administered dose: 200 µl of 1.2×10^8^ PFU/mL of øKp_Pokalde_002 | | | | | | | | | | |
| 1 hour | 34.12 | 27.86 | 60.47 | 34.77 | 83.76 | 60.53 | 113.81 | 61.03 | 57.62 | 133.91 |
| 4 hours | 24.42 | 43.10 | 83.56 | 28.92 | 104.77 | 78.35 | 18.48 | 140.68 | 35.97 | 90.12 |
| 8 hours | 151.57 | 22.23 | 128.21 | 68.00 | 100.51 | 41.11 | 54.69 | 106.94 | 111.55 | 116.27 |
| 24 hours | 53.04 | 100.98 | 60.17 | 99.97 | 76.89 | 138.45 | 88.24 | 72.53 | 109.56 | 90.38 |
| 48 hours | 75.14 | 53.42 | 48.14 | 68.35 | 53.63 | 9.50 | 49.37 | 49.66 | 60.09 | 34.94 |
| 72 hours | 0.00 | 0.00 | 0.00 | 0.00 | 0.00 | 0.00 | 33.07 | 70.50 | 0.00 | 0.00 |
